# Ultra-processed food and incident type 2 diabetes: studying the underlying consumption patterns to unravel the health effects of this heterogeneous food category in the prospective Lifelines cohort

**DOI:** 10.1186/s12916-021-02200-4

**Published:** 2022-01-13

**Authors:** Ming-Jie Duan, Petra C. Vinke, Gerjan Navis, Eva Corpeleijn, Louise H. Dekker

**Affiliations:** 1 Department of Internal Medicine, Division Nephrology (AA52), University Medical Center Groningen, P.O. Box 30 001, 9700RB Groningen, The Netherlands; 2 Department of Epidemiology (FA40), University Medical Center Groningen, P.O. Box 30 001, 9700RB Groningen, The Netherlands; 3 Aletta Jacobs School of Public Health, University of Groningen, P.O. Box 716, 9712GH Groningen, The Netherlands

**Keywords:** Dietary pattern, Epidemiology, Nutrition, Type 2 diabetes, Ultra-processed food

## Abstract

**Background:**

The overall consumption of ultra-processed food (UPF) has previously been associated with type 2 diabetes. However, due to the substantial heterogeneity of this food category, in terms of their nutritional composition and product type, it remains unclear whether previous results apply to all underlying consumption patterns of UPF.

**Methods:**

Of 70,421 participants (35–70 years, 58.6% women) from the Lifelines cohort study, dietary intake was assessed with a food frequency questionnaire. UPF was identified according to the NOVA classification. Principal component analysis (PCA) was performed to derive UPF consumption patterns. The associations of UPF and adherence to UPF consumption patterns with incidence of type 2 diabetes were studied with logistic regression analyses adjusted for age, sex, diet quality, energy intake, alcohol intake, physical activity, TV watching time, smoking status, and educational level.

**Results:**

During a median follow-up of 41 months, a 10% increment in UPF consumption was associated with a 25% higher risk of developing type 2 diabetes (1128 cases; OR 1.25 [95% CI 1.16, 1.34]). PCA revealed four habitual UPF consumption patterns. A pattern high in cold savory snacks (OR 1.16 [95% CI 1.09, 1.22]) and a pattern high in warm savory snacks (OR 1.15 [95% CI 1.08, 1.21]) were associated with an increased risk of incident type 2 diabetes; a pattern high in traditional Dutch cuisine was not associated with type 2 diabetes incidence (OR 1.05 [95% CI 0.97, 1.14]), while a pattern high in sweet snacks and pastries was inversely associated with type 2 diabetes incidence (OR 0.82 [95% CI 0.76, 0.89]).

**Conclusions:**

The heterogeneity of UPF as a general food category is reflected by the discrepancy in associations between four distinct UPF consumption patterns and incident type 2 diabetes. For better public health prevention, research is encouraged to further clarify how different UPF consumption patterns are related to type 2 diabetes.

**Supplementary Information:**

The online version contains supplementary material available at 10.1186/s12916-021-02200-4.

## Background

The magnitude of the worldwide burden of diabetes continues to grow. It is estimated that 578 million people will be living with diabetes globally by the year 2030, approximately 90% of which will be type 2 diabetes [1]. Abundant evidence has shown that adherence to a healthy diet (such as the Mediterranean diet) is crucial to the prevention of type 2 diabetes [2]. However, these dietary patterns studied generally focused on conventional food groups such as fruits and vegetables [3–5]. Recent studies show that higher intake of ultra-processed food (UPF) is associated with higher risk of type 2 diabetes [6, 7]. However, UPF forms a highly heterogeneous food category, especially in terms of their nutritional composition, product types, and contribution to a habitual diet. It is therefore unclear whether previous results that identify total intake of UPF as a single risk factor for type 2 diabetes do apply to all underlying consumption patterns that fall under this “umbrella-term”.

Research on UPF has been facilitated by the development of the NOVA classification. The NOVA classification is a frequently used method to categorize food and drinks based on the nature, extent, and purpose of food processing. The NOVA classification comprises four categories, ranging from un-processed/minimally processed food to UPF [8]. According to the NOVA classification, UPF is mostly formulated from food substances and industrial ingredients that undergo a series of chemical and physical manufacturing processes. The resulting food products are often pre-packed, contain little or no intact (un-processed) food, and are considered microbiologically safe, convenient, and palatable [9].

Since the intake of UPF has substantially increased in most parts of the world over the past decades [10], there is an increasing interest in the potential health impacts of UPF. Prospective cohort studies on the associations between UPF and health so far mostly focused on total intake of UPF. These prospective cohort studies found that higher intake of UPF was associated with higher risks of obesity [11–13], cardiovascular diseases [14–16], cancer [17], mortality [18–20], the metabolic syndrome [21], and type 2 diabetes [6, 7, 22, 23]. Associations established from these studies underline the fact that UPF is not neglectable when studying dietary effects on disease outcomes.

However, an often overlooked virtue of UPF is that it forms a highly heterogeneous food category. Food products considered as UPF are heterogeneous with respect to their nutritional composition, as well as their contribution to a habitual diet, and the context in which they are consumed [24]. For example, according to the frequently used NOVA classification [8], UPF includes pre-packaged bread, a staple food item which in many cultures is consumed with main meals; as well as cakes or fast food, which are consumed more occasionally. Therefore, results from previous studies analyzing UPF as one single food group may not apply to all underlying consumption patterns that fall within this food group. Scientific evidence so far may therefore not be sufficient to formulate evidence-based guidelines and health policies regarding UPF in the battle against type 2 diabetes.

In this study, we first aimed to assess the association between overall UPF intake and incident type 2 diabetes. More importantly, we aimed to identify underlying consumption patterns of UPF and to investigate how they were related to incident type 2 diabetes in a large cohort of Dutch adults.

## Methods

### Cohort design and study population

The Lifelines cohort study is a multidisciplinary prospective population-based cohort study that applies in a unique three-generation design, the health and health-related behaviors of 167,729 persons living in the north of The Netherlands. It employs a broad range of investigative procedures in assessing the biomedical, socio-demographic, behavioral, physical, and psychological factors which contribute to health and disease of the general population.

Participants were included in the study between 2006 and 2013. So far, four follow-up assessment rounds took place, i.e., T1=baseline, median (interquartile) months to follow-up rounds: T2=13 (13-15), T3=25 (23-28), and T4=44 (35-51). Comprehensive physical examinations, biobanking, and questionnaires were conducted at T1 and T4, and follow-up questionnaires (including questions for diabetes status) were issued to participants at T2 and T3. The timeline of data collection of the Lifelines cohort study is presented in Additional file 1: Fig. S1. Before study entry, a signed informed consent form was obtained from each participant. The Lifelines study is conducted according to the principles of the Declaration of Helsinki and approved by the Medical Ethics Committee of the University Medical Center Groningen, The Netherlands (approval number 2007/152). The overall design and rationale of the study have been described in detail elsewhere [25, 26].

Participants aged between 35 and 70 years who were free of diabetes at baseline, and for whom valid dietary intake data was available were included in this study. The ascertainment of prevalent diabetes cases at baseline was based on (1) self-report questionnaires, (2) fasting glucose ≥ 7.0 mmol/L, (3) HbA_1c_ ≥ 48 mmol/mol (6.5%) [27], and (4) medication use on glucose-lowering agents (ATC code A10) [28]. Dietary intake data was considered unreliable when the ratio between reported energy intake and basal metabolic rate (calculated with the Schofield equation [29]) was below 0.50 or above 2.75 (based on the considerations by Goldberg [30]). Furthermore, participants for whom only baseline data was available, or who reported the development of type 1 diabetes or gestational diabetes during the follow-ups, were excluded. In total, 70,421 participants (41,243 women and 29,178 men) were included in the analysis (Additional file 1: Fig. S2).

### Data collection

#### Ascertainment of incident type 2 diabetes

Incident type 2 diabetes was assessed by self-report questionnaires at the two follow-ups (T2, from year 2011 to 2015; and T3, from year 2012 to 2016) and the second assessment (T4, from year 2014 to 2018). Additionally, blood glucose and HbA_1c_ measurements were available at the second assessment (T4). Participants were considered an incident case if they met one of the following criteria: (1) self-reported newly developed type 2 diabetes since last time they filled out a questionnaire, (2) fasting glucose ≥ 7.0 mmol/L, or (3) HbA_1c_ ≥ 48 mmol/mol (6.5%) [27]. However, data on prescribed medication was not available during follow-ups and the precise time of diabetes diagnosis was not documented.

#### Clinical measurements

Blood samples were collected by venipuncture in a fasting state between 8 and 10 am and were further transferred to the Lifelines central laboratory for analysis. Serum levels of glucose and HbA_1c_ were subsequently analyzed. Anthropometric measurements were made by trained research staff following standardized protocols. These measurements were performed without shoes and heavy clothing. BMI was calculated as weight in kilograms divided by the square of height in meters.

#### Dietary assessment

At baseline, dietary consumption was assessed using a validated 110-item semi-quantitative food frequency questionnaire (FFQ), which was designed to assess the food consumption (including alcohol) over the previous month [31]. The questionnaire assessed the frequency of consumption and portion sizes, the latter of which were estimated by fixed portion sizes (e.g., slices of bread, pieces of fruit) and commonly used household measures (e.g., cups, spoons). For insight into the overall diet quality, the food-based Lifelines Diet Score (LLDS) was calculated. This score ranks the relative intake of nine food groups with positive health effects (vegetables, fruit, whole grain products, legumes/nuts, fish, oils/soft margarines, unsweetened dairy, coffee, and tea) and three food groups with negative health effects (red/processed meat, butter/hard margarines, and sugar-sweetened beverages). The development of this score is described in detail elsewhere [32].

#### Categorizing the degree of food processing—the NOVA classification

The NOVA classification was used to categorize all 110 food items into the four proposed categories: (1) un-processed or minimally processed food (e.g., fresh vegetables/fruits, unprocessed meat), (2) processed culinary ingredient (e.g., butter/oil for cooking, sugar, salt), (3) processed food (e.g., canned vegetables/fish, fruits in syrup), and (4) ultra-processed food (e.g., processed meat, soft drinks) [9]. The proportion (weight ratio, %) of intake of UPF in the total weight of food and beverages consumed per day was calculated and was then divided into sex-specific quartiles for further analyses. Using weight ratio of UPF intake accounts for the food that does not provide energy (e.g., artificially sweetened beverages) as well as non-nutritional factors (e.g., additives, by-products during processing). The categorization of the items was verified by four of the authors and can be found in Additional file 1: Table S1.

#### Assessment of other baseline covariates

Age, smoking status, TV watching time, and educational level were assessed by self-administered questionnaires. Smoking status was categorized as never, former, and current smoker. The highest educational level achieved was categorized as (1) low—junior general secondary education or lower (International Standard Classification of Education [ISCED] level 0, 1, or 2); (2) middle—secondary vocational education and senior general secondary education (ISCED level 3 or 4); and (3) high—higher vocational education or university (ISCED level 5 or 6) [33]. The validated Short QUestionnaire to ASsess Health-enhancing physical activity (SQUASH) was used to assess physical activity level [34]. From the SQUASH data, leisure time and commuting physical activities, including sports, at moderate (4.0–6.4 MET) to vigorous (≥ 6.5 MET) intensity (non-occupational moderate-to-vigorous physical activity [MVPA]), were calculated in minutes per week [34]. The variable was categorized by dividing participants who reported any non-occupational MVPA into sex-specific quartiles. For participants who reported zero non-occupational MVPA, the categorical variable was coded as 0.

### Statistical analysis

#### Consumption patterns of ultra-processed food

As UPF is highly heterogeneous on multiple concepts (i.e., nutrient density, nutrient composition, taste, snack or main meal items), it is difficult to create well-founded subgroups. Therefore, instead of using a priori defined subgroups, we used principal component analysis (PCA) to derive underlying consumption patterns of UPF, to obtain real-world insight into the intake of this highly heterogeneous food category. Based on the Scree plot, eigenvalues, and explained variations, four UPF consumption patterns were selected. Thereafter, the derived components were orthogonally rotated to obtain uncorrelated components to enhance interpretability. We selected food items with absolute factor loadings ≥ 0.20 to construct simplified pattern scores while retaining the weight (factor loading) of each selected food item. The simplified UPF consumption pattern scores (hereafter referred to as UPF consumption patterns) were standardized and then divided into sex-specific quartiles for further analyses. Sensitivity analysis was performed by repeating the PCA procedure 3 times on a random half sample.

#### Risk of incident type 2 diabetes

Associations between UPF intake (total intake [continuous or sex-specific quartiles] and UPF consumption patterns [continuous or sex-specific quartiles]) with incident type 2 diabetes were estimated with logistic regression models and results were shown as ORs with 95% confidence intervals. In models where UPF intake was included as a continuous variable (weight ratio), ORs regarding a 10% absolute increment of UPF in the total diet were calculated. In four steps, the analyses were adjusted for (1) age and sex; (2) diet quality (LLDS), total energy intake, and alcohol intake; (3) non-occupational MVPA, TV watching time, smoking status, and educational level; and (4) BMI (continuous). This addition of BMI in the last step aimed to investigate the role of this intermediate factor in the association between UPF and type 2 diabetes. Additionally, the possibility of effect modification by sex was tested by including the interaction-term for sex and UPF intake in the models. To account for missing covariates, multiple imputation by chained equations was performed to deal with missing data for non-occupational MVPA (proportion of missing 6.5%), TV watching time (proportion of missing 0.6%), smoking status (proportion of missing 0.6%), and educational level (proportion of missing 0.4%).

We performed several sensitivity analyses to test the robustness of our results. First, analyses were performed using energy-adjusted UPF intake. Second, sensitivity analyses on missing data were performed by complete case analysis. Moreover, we excluded participants who were lost to follow-up after 24 months, in an attempt to address the possible reverse causation caused by short follow-up time.

#### Post hoc analysis—baseline diabetes risk and ultra-processed food consumption patterns

Individuals’ awareness of elevated diabetes risk may have influenced individuals’ dietary behaviors at baseline. Therefore, linear regression models were performed to investigate whether type 2 diabetes risk at baseline, as calculated with the PROCAM risk algorithm (Additional file 1: Table S2) [35], was associated with the total intake of UPF and distinctive UPF consumption patterns. In the linear regression models, the total intake of UPF or the UPF consumption pattern scores were set as dependent variable one by one. The analyses were additionally adjusted for the same covariates as described above, except for energy intake and BMI. Energy intake was not considered to be a confounding factor, and BMI was not included due to its high correlation with the PROCAM diabetes risk algorithm (Pearson correlation coefficient = 0.835).

## Results

Baseline characteristics across quartiles of UPF consumption are shown in Table 1. In the total study population, the median contribution of UPF to the total diet was 34.9 weight% (Additional file 1: Fig. S3). Of all UPF groups, staple/starchy food and cereals like sliced bread or granola (22.1%), non-cheese dairy products like chocolate milk and ice cream (13.7%), and sugary beverages like lemonade or ice tea (9.7%) contributed most to the overall intake of UPF (median weight% of total UPF, Additional file 1: Table S3). In general, with increasing quartiles of UPF consumption, participants tended to be younger, have higher BMI, have lower type 2 diabetes risk scores, be less physically active, have worse overall diet quality, consume less alcohol, smoke less, be less highly educated, and spend more time on watching TV.

**Table 1:** Baseline characteristics of study participants according to sex-specific quartiles of ultra-processed food consumption (*n* = 70,421)^a, b^

	Quartiles of ultra-processed food consumption				
	First (***n*** = 17,604)	Second (***n*** = 17,606)	Third (***n*** = 17,606)	Fourth (***n*** = 17,606)	Total (***n*** = 70,421)
Age, years	52.3±9.1	50.2±8.8	48.3±8.4	45.7±7.6	49.1±8.8
Sex, %					
Women	58.6	58.6	58.6	58.6	58.6
Men	41.4	41.4	41.4	41.4	41.4
Ultra-processed food intake, weight%	23.7 (20.3, 26.0)	31.6 (29.0, 34.1)	38.4 (35.6, 40.5)	48.7 (45.2, 53.9)	34.9 (28.1, 42.7)
Lifeline diet score	28.6±5.1	25.4±4.7	22.8±4.6	19.2±4.7	24.0±5.9
Total energy intake, kcal/day	1811±520	2032±543	2150±579	2261±647	2063±598
Total alcohol intake, grams/day	6.2 (1.4, 12.1)	5.8 (1.3, 11.4)	4.4 (1.0, 10.4)	2.9 (0.4, 9.9)	4.7 (0.9, 11.2)
Fasting glucose, mmol/L	4.96±0.51	4.96±0.50	4.96±0.50	4.97±0.51	4.96±0.50
HbA_1c_, %	5.56±0.30	5.56±0.30	5.54±0.30	5.54±0.30	5.55±0.30
HbA_1c_, mmol/mol	37.3±3.2	37.2±3.2	37.1±3.3	37.0±3.3	37.2±3.3
BMI, kg/m^2^	25.6±3.8	25.6±3.9	26.2±4.0	26.7±4.5	26.2±4.1
Highest tertile of PROCAM diabetes risk algorithm, %	37.2	33.2	31.9	30.4	33.2
MVPA, minutes/week^c^	240 (90, 420)	210 (80, 380)	180 (60, 360)	150 (60, 330)	190 (60, 365)
Educational level, %					
Low	28.4	29.5	30.5	33.1	30.4
Middle	34.8	37.7	40.1	42.8	38.9
High	36.2	32.4	29.0	23.7	30.3
Smoking status, %					
Never	39.8	43.6	45.4	48.7	44.4
Former	44.2	40.0	36.8	30.6	37.9
Current	15.4	15.8	17.4	20.0	17.2
TV watching time, hours/day	2.4±1.3	2.4±1.3	2.5±1.2	2.6±1.4	2.5±1.3

^a^Data are expressed as unadjusted mean ± standard deviation for age, Lifelines diet score (no unit), total energy intake, fasting glucose, HbA_1c_, BMI, and TV watching time; data are expressed as median (interquartile) for ultra-processed food intake (weight percentage), total alcohol intake, and MVPA; data are expressed as observed percentage for sex, highest tertile of PROCAM diabetes risk algorithm, educational level, and smoking status

^b^Tests for significant differences in baseline characteristics (except for sex) across quartiles of ultra-processed food consumption were performed using Kruskal-Wallis test or *χ*^2^ test for proportion, as appropriate; *P* < 0.001 for all baseline characteristics except for fasting glucose *P* = 0.019

^c^MVPA denotes non-occupational moderate-to-vigorous physical activity level

### Overall consumption of ultra-processed food and risk of incident type 2 diabetes

Table 2 shows the association between consumption of UPF and the risk of incident type 2 diabetes. Among 70,421 participants included in the analysis, we identified 1128 cases (550 female cases and 578 male cases, Additional file 1: Fig. S2) of type 2 diabetes during a median follow-up of 41 months. A significant positive association between the overall consumption of UPF and incident type 2 diabetes was observed. Per 10% absolute increment intake of UPF, participants had 33% higher odds of incident type 2 diabetes (OR 1.33 [95% CI 1.26, 1.41], *P* < 0.001, model 1, sex and age adjusted). This association remained significant after additional adjustment for diet quality and other covariates (OR 1.25 [95% CI 1.16, 1.34], *P* < 0.001, model 3). Additional adjustment for BMI further explained part of the association (OR 1.17 [95% CI 1.09, 1.26], model 4). When comparing the highest versus the lowest quartile of UPF consumption, participants in the highest quartile had an 80% higher odds of incident type 2 diabetes (OR Q4 versus Q1 1.80 [95% CI 1.47, 2.20], *P-*trend < 0.001, model 3). We did not find evidence of effect modification by sex (*P*_interaction_ > 0.05). Sensitivity analyses on missing data showed similar results (Additional file 1: Table S4). Excluding participants who only had 24 months of follow-up also yielded similar results (Additional file 1: Table S5). In addition, results from energy-adjusted intake of UPF were basically unchanged (Additional file 1: Table S6).

**Table 2:** Associations between consumption of ultra-processed food and incident type 2 diabetes

	Quartiles of ultra-processed food consumption						
	First	Second	Third	Fourth	***P***-trend	Continuous^a^	***P*** value
Cases/population	255/17,604	247/17,606	272/17,605	354/17,606		1128/70,421	
Incidence, %	1.4	1.4	1.5	2.0		1.6	
Model 1^b^	1	1.11 (0.93, 1.33)	1.39 (1.17, 1.65)	2.17 (1.83, 2.58)	< 0.001	1.33 (1.26, 1.41)	< 0.001
Model 2^c^	1	1.08 (0.90, 1.30)	1.30 (1.07, 1.57)	1.87 (1.52, 2.30)	< 0.001	1.27 (1.18, 1.36)	< 0.001
Model 3^d^	1	1.08 (0.90, 1.30)	1.28 (1.06, 1.55)	1.80 (1.47, 2.20)	< 0.001	1.25 (1.16, 1.34)	< 0.001
Model 4^e^	1	1.04 (0.87, 1.26)	1.20 (0.99, 1.45)	1.56 (1.27, 1.92)	< 0.001	1.17 (1.09, 1.26)	< 0.001

^a^Continuous model indicates OR (95% CI) for an absolute increment of 10% consumption of ultra-processed food in the total diet

^b^Model 1: OR (95% CI) derived from multivariate logistic regression models adjusted for age and sex, *n* = 70,421

^c^Model 2: OR (95% CI) derived from multivariate logistic regression models adjusted for Model 1 covariates plus Lifelines diet score, total energy intake, and alcohol intake, *n* = 70,421

^d^Model 3: OR (95% CI) derived from multivariate logistic regression models adjusted for Model 2 covariates plus smoking status, educational level, non-occupational moderate-to-vigorous physical activity level, and TV watching time, *n* = 70,418

^e^Model 4: OR (95% CI) derived from multivariate logistic regression models adjusted for Model 3 covariates plus BMI, *n* = 70,403

### UPF consumption patterns and incident type 2 diabetes

To identify habitual consumption patterns of UPF, we performed PCA analysis and selected four UPF consumption patterns. These four patterns explained 15.5% of the total variance of UPF intake. Additional file 1: Table S7 shows the factor loadings of UPF products within their corresponding consumption patterns. Briefly, these four patterns were (1) warm savory snack pattern, characterized by high intake of fried snacks, fries, and snack sauce; (2) cold savory snack pattern, characterized by high intake of cheese, deli meat, and savory spreads for crackers or French bread; (3) traditional Dutch cuisine pattern, characterized by high intake of main meal items typical for the Dutch culture, such as sliced bread, lunch meat, and gravy; and (4) sweet snack pattern, characterized by high intake of sweet biscuits/cookies, pastries, and chocolate. Explained variance was highest for the warm savory snack pattern (5.0%) and lowest for the cold savory snack pattern (3.3%). Baseline characteristics across different UPF consumption patterns (highest quartiles) are shown in Additional file 1: Table S8. Similar UPF consumption patterns were identified when analzying the random half sample (Additional file 1: Table S9).

Associations between UPF habitual consumption patterns and incident type 2 diabetes are shown in Table 3. For the ORs treating consumption pattern scores as a continuous variable, the warm savory snack pattern (OR 1.15 [95% CI 1.08, 1.21], *P* < 0.001) and the cold savory snack pattern (OR 1.16 [95% CI 1.09, 1.22], *P* < 0.001) were positively associated with incident type 2 diabetes (model 3). For the traditional Dutch cuisine pattern, no significant association was found with incident type 2 diabetes (OR 1.05 [95% CI 0.97, 1.14], *P* = 0.207, model 3). Oppositely, higher adherence to the sweet snack pattern was negatively associated with incident type 2 diabetes (OR 0.82 [95% CI 0.76, 0.89], *P* < 0.001, model 3). Results were consistent when comparing the highest quartile with the lowest quartile of the consumption pattern scores. Additional adjustment for baseline BMI (model 4) led to minor attenuation of all associations, except for the warm savory snack pattern. For the latter, the ORs were moderately attenuated and became insignificant, but were still positively associated with higher risk of incident type 2 diabetes (OR 1.07 [95%CI 1.00, 1.14], *P* = 0.057; OR Q4 versus Q1 1.17 [95%CI 0.96, 1.44], *P-*trend = 0.097). Sensitivity analysis on missing data (complete case analysis) yielded similar results (Additional file 1: Table S4). Results are basically unchanged when excluding participants who were lost to follow-up after 24 months (Additional file 1: Table S5).

**Table 3:** Associations between ultra-processed food consumption pattern scores and incident type 2 diabetes

Consumption patterns scores	Models	Quartiles of consumption pattern scores of ultra-processed food						
		First	Second	Third	Fourth	***P***-trend	Continuous	***P*** value
Warm savory snack pattern	Cases/population	291/17,561	272/17,649	273/17,605	292/17,606			
	Model 1^a^	1	1.15 (0.97, 1.37)	1.42 (1.19, 1.69)	1.82 (1.52, 2.18)	< 0.001	1.22 (1.16, 1.27)	< 0.001
	Model 2^b^	1	1.13 (0.95, 1.34)	1.36 (1.14, 1.64)	1.74 (1.43, 2.12)	< 0.001	1.22 (1.16, 1.29)	< 0.001
	Model 3^c^	1	1.07 (0.90, 1.27)	1.23 (1.02, 1.48)	1.43 (1.17, 1.75)	< 0.001	1.15 (1.08, 1.21)	< 0.001
	Model 4^d^	1	1.02 (0.86, 1.22)	1.11 (0.92, 1.34)	1.17 (0.96, 1.44)	0.097	1.07 (1.00, 1.14)	0.057
Traditional Dutch cuisine pattern	Cases/population	276/17,605	282/17,605	301/17,605	269/17,606			
	Model 1^a^	1	1.01 (0.85, 1.19)	1.11 (0.94, 1.31)	1.06 (0.89, 1.26)	0.332	1.03 (0.97, 1.10)	0.330
	Model 2^b^	1	1.06 (0.89, 1.26)	1.19 (0.99, 1.43)	1.15 (0.94, 1.42)	0.113	1.07 (0.99, 1.15)	0.101
	Model 3^c^	1	1.05 (0.88, 1.25)	1.16 (0.96, 1.39)	1.11 (0.90, 1.37)	0.192	1.05 (0.97, 1.14)	0.207
	Model 4^d^	1	1.03 (0.87, 1.23)	1.11 (0.92, 1.33)	1.07 (0.87, 1.31)	0.411	1.03 (0.95, 1.11)	0.476
Sweet snack pattern	Cases/population	400/17,605	273/17,605	231/17,605	224/17,606			
	Model 1^a^	1	0.68 (0.58, 0.79)	0.58 (0.49, 0.68)	0.60 (0.50, 0.70)	< 0.001	0.82 (0.76, 0.89)	< 0.001
	Model 2^b^	1	0.66 (0.57, 0.78)	0.55 (0.47, 0.66)	0.53 (0.44, 0.64)	< 0.001	0.79 (0.72, 0.86)	< 0.001
	Model 3^c^	1	0.69 (0.59, 0.81)	0.60 (0.50, 0.71)	0.59 (0.49, 0.71)	< 0.001	0.82 (0.76, 0.89)	< 0.001
	Model 4^d^	1	0.75 (0.64, 0.88)	0.68 (0.57, 0.81)	0.69 (0.57, 0.84)	< 0.001	0.87 (0.80, 0.94)	0.001
Cold savory snack pattern	Cases/population	292/17,605	266/17,605	289/17,605	281/17,606			
	Model 1^a^	1	0.98 (0.83, 1.16)	1.10 (0.93, 1.30)	1.08 (0.92, 1.28)	0.188	1.09 (1.02, 1.15)	0.007
	Model 2^b^	1	1.04 (0.88, 1.24)	1.20 (1.01, 1.43)	1.22 (1.03, 1.46)	0.010	1.13 (1.06, 1.20)	< 0.001
	Model 3^d^	1	1.07 (0.91, 1.27)	1.27 (1.07, 1.51)	1.33 (1.12, 1.59)	< 0.001	1.16 (1.09, 1.22)	< 0.001
	Model 4^d^	1	1.04 (0.87, 1.23)	1.20 (1.00, 1.42)	1.20 (1.00, 1.44)	0.020	1.11 (1.04, 1.18)	0.001

^a^Model 1: OR (95% CI) derived from multivariate logistic regression models adjusted for age and sex, *n* = 70,421

^b^Model 2: OR (95% CI) derived from multivariate logistic regression models adjusted for Model 1 covariates plus Lifelines diet score, total energy intake, and alcohol intake, *n* = 70,421

^c^Model 3: OR (95% CI) derived from multivariate logistic regression models adjusted for Model 2 covariates plus smoking status, educational level, non-occupational moderate-to-vigorous physical activity level, and TV watching time, *n* = 70,418

^d^Model 4: OR (95% CI) derived from multivariate logistic regression models adjusted for Model 3 covariates plus BMI, *n* = 70,403

### Baseline diabetes risk and ultra-processed food consumption patterns

To explore how diet may be dependent on the baseline health condition, the estimated diabetes risk score at baseline was related to the total intake of UPF and four UPF consumption patterns (Table 4). The results showed that baseline type 2 diabetes risk was positively associated with the total UPF intake, as well as the warm savory snack pattern and the cold savory snack pattern, but negatively associated with the traditional Dutch cuisine pattern and the sweet snack pattern. The strongest association was found for the sweet snack pattern (*β* = − 0.104 [95% CI − 0.113, − 0.094], *P* < 0.001), which indicates that those with high diabetes risk scores at baseline had lower adherence to the sweet UPF pattern. Results from complete case analysis are basically unchanged (Additional file 1: Table S10).

**Table 4:** Associations of ultra-processed food intake and its consumption patterns with type 2 diabetes risk at baseline^a^

Ultra-processed food consumption (patterns)	Standardized beta-coefficients^b^
Total ultra-processed food intake	0.052 (0.045, 0.059)
Warm savory snack pattern	0.091 (0.082, 0.101)
Traditional Dutch cuisine pattern	− 0.032 (− 0.041, − 0.023)
Sweet snack pattern	− 0.104 (− 0.113, − 0.094)
Cold savory snack pattern	0.041 (0.032, 0.050)

^a^Type 2 diabetes risk at baseline was assessed by PROCAM diabetes risk algorithm (Supplementary Table S2)

^b^Standardized beta-coefficients (95% CI) derived from multivariate linear regression models adjusted for age, sex, Lifelines diet score, alcohol intake, smoking status, educational level, non-occupational moderate-to-vigorous physical activity level, and TV watching time, all *P* values < 0.001, *n* = 70,085

## Discussion

In this large population-based cohort study, the overall consumption of UPF was associated with a higher risk of type 2 diabetes, independent of overall diet quality and energy intake. We illustrated the importance of considering the heterogeneity of UPF when studying its health effects, as associations with incident type 2 diabetes varied across different patterns of UPF consumption. A positive association with incident type 2 diabetes was found for both warm savory snack and cold savory snack UPF consumption patterns, while a negative association was found for sweet snack UPF pattern. On the other hand, the absence of a clear association between diabetes risk and the traditional Dutch cuisine UPF pattern, which was high in main meal food items, suggests that not all types of UPF are necessarily detrimental to health.

Over the past few years, scientific interests and public awareness on UPF have risen substantially [36, 37]. So far, four studies have investigated the association of UPF with type 2 diabetes [6, 7, 22, 23]. Our results provide an independent confirmation of the association between UPF intake and incident type 2 diabetes in a different population setting. When comparing our results to those from the French NutriNet-Santé cohort and the UK Biobank cohort in which similar methods were used, the extent to which UPF contributed to the habitual diet differed considerably. The mean weight percentage of UPF in the diet was 35.9% in this Dutch cohort, versus 15.4% in the French cohort [6] and 22.1% in the British cohort [7]. Nevertheless, the reported hazard ratio of 1.15 in the previous French study and 1.12 in the UK Biobank study, regarding each 10 percent increment in the proportion of UPF in the diet, were comparable to our OR of 1.17 in our fourth model, adjusting for comparable potential confounding factors. In addition, it is noteworthy that in all three studies, associations were independent of the overall diet quality as well as total energy intake. This consolidates the potential role of UPF as an independent dietary factor in the development of type 2 diabetes. More importantly, it emphasizes that eating an otherwise healthy diet may not fully compensate for the detrimental effects of UPF.

Notwithstanding the high heterogeneity among different types of UPF, previous studies on the health consequences of UPF mainly focused on its overall intake. To our knowledge, the current study is the first that investigated the relation of overall intake and consumption patterns of UPF with incident type 2 diabetes in a large population-based sample. Our findings emphasize that it is crucial to consider various habitual UPF consumption patterns and their unique food groups when studying their health effects. In line with overall UPF intake, both the warm savory snack and the cold savory snack UPF patterns were associated with higher risks of type 2 diabetes. Results deviated for the traditional Dutch cuisine pattern and the sweet snack pattern, as the associations with type 2 diabetes were absent for the first, and inverse for the latter. The absence of an association for the traditional Dutch cuisine pattern illustrates that the detrimental effects of UPF may not be solely due to the degree of food processing. As UPF forms a highly heterogeneous food category, it is also important to consider their nutritional quality [38]. For instance, a key food product in the traditional Dutch cuisine pattern was sliced bread. Despite mostly being ultra-processed, approximately 70% of the sliced bread consumed in The Netherlands is brown bread (made with a mixture of whole-wheat and white flour) or whole-wheat bread, and therefore often high in fiber and micronutrients. Higher intake of fiber and whole-wheat products was found to be associated with lower risk of type 2 diabetes [39]. On the other hand, the UPF products identified in these two savory snack patterns are generally high in salt and fat and are often energy dense. It is conceivable that they may increase diabetes risk through metabolic disturbances, such as elevated blood pressure and lipid abnormality [40, 41]. Therefore, a cautious interpretation of the health effects of UPF is warranted. More specifically, their effects on health may be determined by more than the level of food processing alone, which makes that not all types of UPF are necessarily detrimental to health.

Despite remaining statistically significant, our observation that estimates for the associations between UPF intake and type 2 diabetes were clearly attenuated when additionally adjusting for BMI, illustrating that BMI plays a role in the studied association. This role, however, may be two-fold, as BMI may be both a confounding and a mediating factor. Individuals with higher baseline BMI appeared to have higher total UPF intake, as well as a higher risk of type 2 diabetes, showing its confounding property. However, since previous prospective studies have illustrated that UPF is a risk factor for obesity [11–13], higher intake of UPF may also increase type 2 diabetes risk through an increase in body weight, which illustrates the potential mediating role of BMI in the association studied. However, we also could not rule out the possibility of residual confounding, even in our analysis various involving covariates (including demographic, lifestyle, and socio-economic factors) were adjusted. Future studies, preferably in the form of randomized controlled trials, are required to help disentangle the role of BMI in the relationship between UPF and health.

Our finding that higher adherence to the sweet snack UPF pattern was associated with lower risk of incident type 2 diabetes was counterintuitive. Previous evidence indicates that the intake of dietary sugar from food and beverages was associated with weight gain and obesity, and may thus contribute to the risk of type 2 diabetes [42, 43]. Nonetheless, a study in EPIC (European Prospective Investigation into Cancer and Nutrition) also found that non-consumers of cakes and cookies had a higher risk of type 2 diabetes [44]. To assess for possible reverse causation, we performed a post hoc analysis to evaluate whether individuals’ baseline type 2 diabetes risk score was involved in this unexpected finding. As shown, a higher PROCAM diabetes risk score at baseline was associated with lower adherence to the sweet snack UPF pattern. Those with a high risk of type 2 diabetes could have been made aware of their situation through opportunistic screening by general practitioners, public health campaigns, or family history of the disease. Hence, awareness of high type 2 diabetes risk may have driven participants to avoid products that are high in sugar.

From a public health point of view, this can be perceived as a positive message, suggesting that public health initiatives to inform the public on the importance of a healthy diet in the prevention of chronic diseases, such as type 2 diabetes, did come across. In addition, the fact that the inverse association with baseline type 2 diabetes risk observed for the sweet snack pattern may be related to the layman’s term for type 2 diabetes, which is “sugar disease” in Dutch and several other languages. Although there is still some scientific uncertainty as to whether all types of sugar intake are associated with risk of type 2 diabetes [45–48], limiting the consumption of energy dense, sugar-rich foods will be likely to benefit health, not only by reducing the risk of diabetes, but obesity and cardiovascular diseases as well [48]. Furthermore, it is worth noticing that the adherence to both two savory UPF patterns was higher among individuals with higher diabetes risk scores at baseline, and both patterns were also associated with a higher risk of incident type 2 diabetes. Future research, preferably in the form of randomized controlled trials, is needed to confirm a detrimental effect on glucose homeostasis from both sugary and savory UPF items. A subsequent challenge would then be to create further awareness that it is not only sugary products, but also other kinds of UPF, which may be associated with higher diabetes risk. This could also bear relevance to prevention strategies not only by recommendations for health behaviors, but also by recommendations for product reformulation [49].

Strengths of this study include the large sample size, which yields a strong statistical power. In addition, our study is the first that thoroughly investigated the habitual consumption patterns of UPF using PCA. The empirical consumption patterns identified reflected not only nutritional properties of UPF, but also its behavioral drivers, which provide a distinct added value over the nutritional information of the NOVA classification and strengthen the real-world robustness of the results of this study [50]. Nevertheless, it should be noted that the four consumption patterns analyzed in total only explained 15.5% of the total variance of UPF intake, which inevitably left a certain proportion of the UPF consumption pattern information uncaptured. It is conceivable that this seemingly low explained variance is attributed to our large study sample size. Secondly, this may also be attributed to the fact that we did not apply massive food groupings in dietary pattern analysis (i.e., combining the intake of several food products into one, such as treating all sorts of cheeses as one single food product) [50, 51], which in fact facilitates our study objective for the disentanglement of the consumption patterns of this highly heterogeneous food category. On the other hand, our 15.5% explained variance is comparable with previous studies using PCA and did offer us informative insights into the real-world eating patterns, especially considering our cohort setting [51, 52]. We encourage future studies to further explore the UPF consumption patterns in a different population setting.

Furthermore, there are also several other limitations that should be noted. First of all, the FFQ used in this study was designed to assess the intake of major food groups, energy, and macronutrients. The aim to assess energy intake resulted in good coverage of energy dense food, including many kinds of UPF. However, since the FFQ was not designed to assess the intake of UPF, it included questions that covered the intake of food items with varying levels of processing, inevitably leading to some misclassification. Second, misclassification could also occur in the ascertainment of type 2 diabetes cases, since at T2 and T3 only self-reported data was available. However, as most cases were identified by objective laboratory measurements at T4, this limitation is not expected to influence our results. Third, the exact time of diagnosis of diabetes cases was not collected in the Lifelines study which unfortunately reduced the suitability of our data for survival analyses. Nevertheless, considering the low event rate and the relatively short follow-up time, logistic regression analysis may provide similar estimates for the effect sizes [53, 54]. We therefore used logistic regression analysis instead. Furthermore, the use of self-reported questionnaires such as FFQ might lead to misreporting due to social desirability or recall bias. Finally, we illustrated that some reverse causation could be involved in the results of this study, despite our prospective design.

## Conclusions

In conclusion, this study illustrated that the heterogeneity of UPF as a general food category is also reflected by the discrepancy in associations of four distinct UPF consumption patterns and incident type 2 diabetes. The positive associations of the warm savory snack and the cold savory snack UPF consumption patterns with incident type 2 diabetes suggest that savory UPF may be a suitable target for future public health initiatives for type 2 diabetes prevention. More importantly, since UPF consumption was associated with type 2 diabetes risk independent of overall diet quality, eating an otherwise healthy diet may not fully compensate for the detrimental effects of UPF. Therefore, in addition to promoting consumption of healthy food products, active discouragement of unhealthy food products such as savory UPF should be considered as part of diabetes prevention strategies. In addition, considering the intricate role of BMI in the relationships between UPF and health, it is of equal importance to consider weight management in public health promotion, in addition to the discouragement of UPF consumption. Further research on UPF subgroups and its underlying consumption patterns is encouraged to allow for a better understanding of the health effects of this highly heterogeneous food category, which will also facilitate the integration of UPF into dietary assessment tools and recommendations.

## Supplementary Information

**Additional file 1: Figures S1-S3** and **Tables S1-S10.** **Fig. S1.** Timeline of data collection of the Lifelines cohort study. **Fig. S2.** Study flow chart for exclusions and diagnosis of type 2 diabetes cases. **Fig. S3.** Overall contribution of ultra-processed food to total diet. **Table S1.** Categorization of food-items in ultra-processed food categories. **Table S2.** Calculation of PROCAM diabetes risk score algorithm. **Table S3.** Contribution of ultra-processed food sub-groups to overall intake of ultra-processed food. **Table S4.** Sensitivity analysis on missing data - ultra-processed food intake and incident type 2 diabetes. **Table S5.** Sensitivity analysis on excluding participants with only 24 months of follow-up. **Table S6.** Sensitivity analysis using energy-adjusted intake of ultra-processed food. **Table S7.** Factor loadings of ultra-processed food products within their corresponding consumption patterns. **Table S8.** Baseline characteristics across different ultra-processed food consumption patterns. **Table S9.** Sensitivity analysis of ultra-processed food consumption patterns using random half sample. **Table S10.** Sensitivity analysis on missing data - ultra-processed food intake/consumption patterns and diabetes risk at baseline.

## Data Availability

The manuscript is based on the data from the Lifelines cohort study. Lifelines adheres to standards for data availability. The data catalog of the Lifelines cohort study is publicly accessible at www.lifelines.nl. All international researchers can obtain data at the Lifelines research office (research@lifelines.nl), for which a fee is required. The Lifelines research system allows access for reproducibility of the study results.

## Abbreviations

FFQ: Food frequency questionnaire
LLDS: Lifelines diet score
MVPA: Moderate-to-vigorous physical activity
PCA: Principal component analysis
UPF: Ultra-processed food
