# Supplementary material for: Ultra-processed food and incident type 2 diabetes: studying the underlying consumption patterns to unravel the health effects of this heterogeneous food category in the prospective Lifelines cohort
Source: BMC Med. 2022 Jan 13;20:7. doi: 10.1186/s12916-021-02200-4 (PMC8756643; doi:10.1186/s12916-021-02200-4)
Supplement: Supplementary file 1 — Additional file 1: Figures S1-S3 and Tables S1-S10. Fig. S1. Timeline of data collection of the Lifelines cohort study. Fig. S2. Study flow chart for exclusions and diagnosis of type 2 diabetes cases. Fig. S3. Overall contribution of ultra-processed food to total diet. Table S1. Categorization of food-items in ultra-processed food categories. Table S2. Calculation of PROCAM diabetes risk score algorithm. Table S3. Contribution of ultra-processed food sub-groups to overall intake of ultra-processed food. Table S4. Sensitivity analysis on missing data - ultra-processed food intake and incident type 2 diabetes. Table S5. Sensitivity analysis on excluding participants with only 24 months of follow-up. Table S6. Sensitivity analysis using energy-adjusted intake of ultra-processed food. Table S7. Factor loadings of ultra-processed food products within their corresponding consumption patterns. Table S8. Baseline characteristics across different ultra-processed food consumption patterns. Table S9. Sensitivity analysis of ultra-processed food consumption patterns using random half sample. Table S10. Sensitivity analysis on missing data - ultra-processed food intake/consumption patterns and diabetes risk at baseline. [file 12916_2021_2200_MOESM1_ESM.docx]

**Ultra-processed food and incident Type 2 Diabetes: studying the underlying consumption patterns to unravel the health effects of this heterogeneous food category in the prospective Lifelines cohort**

**Additional File 1**

Ming-Jie Duan*^1^; Petra C. Vinke*^2^; Gerjan Navis^1^; Eva Corpeleijn^2^; Louise H. Dekker^1,3^.

Corresponding author: Ming-Jie Duan, [m.duan@umcg.nl](mailto:m.duan@umcg.nl)

^1^ University Medical Center Groningen, Department of Internal Medicine, Division Nephrology (AA52), P.O. Box 30 001, 9700 RB Groningen, The Netherlands

^2^ University Medical Center Groningen, Department of Epidemiology (FA40), P.O. Box 30 001, 9700 RB Groningen, The Netherlands

^3^ Aletta Jacobs School of Public Health, University of Groningen, Groningen, The Netherlands

**Contributed equally*

**Additional file 1: Fig. S1** - Timeline of data collection of the Lifelines cohort study


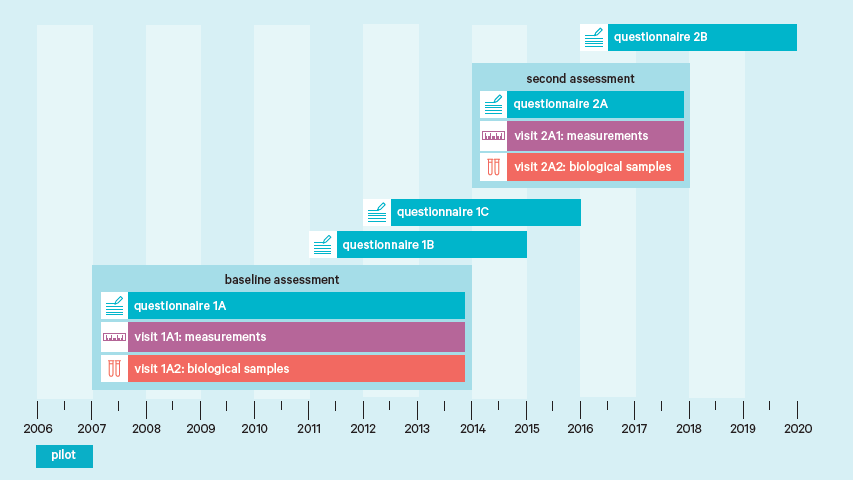


|  | Baseline assessment T1 | Follow-up T2 (questionnaire 1B) | Follow-up T3 (questionnaire 1C) | Second assessment T4 |
| --- | --- | --- | --- | --- |
| Blood samples | **Yes** | No | No | **Yes** |
| Questionnaires for diabetes status | **Yes** | **Yes** | **Yes** | **Yes** |
| Dietary assessment (food frequency questionnaires) | **Yes** | No | No | Yes |
| Anthropometry | **Yes** | No | No | Yes |
| Other covariates | **Yes** | No | No | Yes |

(1) Detailed information can be found at: <https://www.lifelines.nl/researcher/data-and-biobank>

(2) For this study, blood samples collected at T1 and T4 and questionnaires for diabetes status collected in all follow-up visits were used to identify diabetes cases; dietary assessment at baseline was used to quantify ultra-processed food intake; anthropometry and other covariates assessed at baseline were used as covariates adjusted in the analysis.

**Additional file 1: Fig. S2** – Study flow chart for exclusions and diagnosis of type 2 diabetes cases


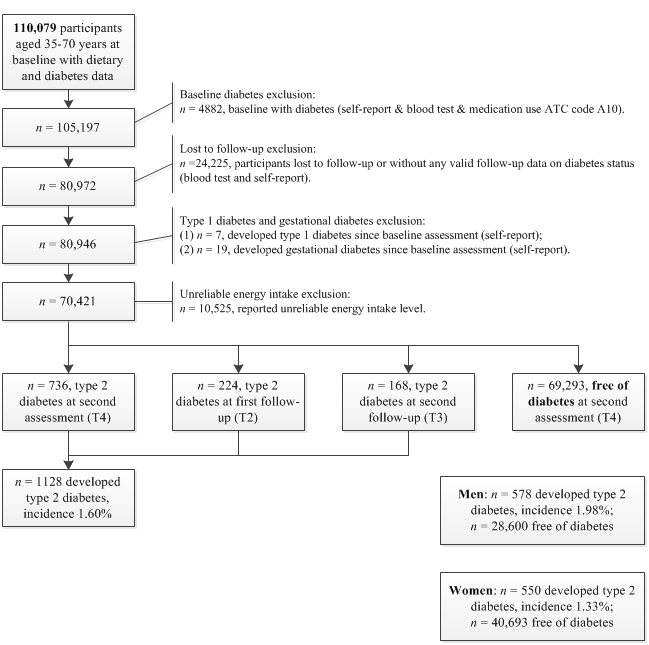


**Additional file 1: Fig. S3** - Distribution of ultra-processed food consumption in the diet (weight percentage, %)


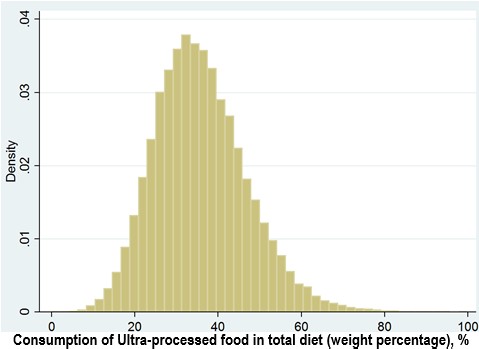


Study sample size *n* = 70 421, mean 35.9%, standardized deviation 11.1%, median 34.9%, interquartile (28.1%, 42.7%).

**Additional file 1: Table S1** - Food items considered as ultra-processed food according to the NOVA-classification

| **Ultra-processed food groups** | **Food products** |
| --- | --- |
| Cheese | 20+/30+/40+/48+ cheese or spreadable cheese, snack cheese, cheese with warm meals, foreign cheese, cream cheese |
| Dairy products (except cheese) | Chocolate milk, yogurt drinks, ready-to-eat porridge, whipped cream, ice-cream, quark and fruit quark, fruit yogurt, full-fat custard, other types of flavored dairy products |
| Meat, fish | Luncheon meat, smoked sausages, deli meat, bacon slices, ham sausages, deep-fried whiting in dough, other types of processed meat |
| Savory snacks | Croquettes, minced meat hotdogs, sausage rolls, potato chips, salty biscuits, other types of warm savory snacks |
| Sweets, candies, and other sugary products | Apple sauce, small cookies/biscuits, cakes, pastries, pies, breakfast biscuits, cookie bars, nutritional biscuits, candy bars, chocolates, candies |
| Savory spreads, sauces, and salad toppings for bread | Salad (spreads) on bread/baguettes, sandwich spreads, other kind of savory spreads on bread/baguettes, gravy, pasta sauce, mushroom sauce, sate sauce, sauce for fries, regular mayonnaise, low-fat mayonnaise, salad dressing, other non-red sauces |
| Sweet spreads | Peanut butter, chocolate spreads, other types of sweet spreads |
| Ready-to-eat meals | Chinese/Indonesian dishes, other types of ready-to-eat meals |
| Fast food | Meals from fast-food restaurants, pizza |
| Staple/starchy food, and cereals | Sliced bread, rusk, crisp bread, croissants, fries, muesli, granola, cereals for the preparation of porridges |
| Soup | Pre-packed soup |
| Sugary beverages | Sugary beverages, lemonade with sugar, juice |
| Spreadable butter | Spreadable butter, margarine, low fat margarine |
| Other solid foods | Coffee creamer |
| Other liquid foods | Alcohol free beer, breakfast drink, light soft drinks, lemonade without sugar |

**Additional file 1: Table S2** - Calculation of diabetes risk at baseline by PROCAM diabetes risk algorithm and baseline characteristics of predictors in PROCAM diabetes risk algorithm according to sex-specific quartiles of ultra-processed food consumption (*n* = 70 421)*

|  | **Quartiles of ultra-processed food consumption** | | | |  |
| --- | --- | --- | --- | --- | --- |
|  | **First (*n* = 17 604)** | **Second (*n* = 17 606)** | **Third (*n* = 17 606)** | **Fourth (*n* = 17 606)** | **Total (*n* = 70 421)** |
| Age, years | 52.3±9.1 | 50.2±8.8 | 48.3±8.4 | 45.7±7.6 | 49.1±8.8 |
| Ultra-processed food intake, % | 23.7 (20.3, 26.0) | 31.6 (29.0, 34.1) | 38.4 (35.6, 40.5) | 48.7 (45.2, 53.9) | 34.9 (28.1, 42.7) |
| PROCAM diabetes risk value | -14.27±0.57 | -14.33±0.57 | -14.35±0.58 | -14.38±0.60 | -14.33±0.58 |
| Highest tertile of PROCAM diabetes risk algorithm, % | 37.2 | 33.2 | 31.9 | 30.4 | 33.2 |
| Fasting glucose, mmol/L | 4.96±0.51 | 4.96±0.50 | 4.96±0.50 | 4.97±0.51 | 4.96±0.50 |
| BMI, kg/m^2^ | 25.6±3.8 | 25.6±3.9 | 26.2±4.0 | 26.7±4.5 | 26.2±4.1 |
| HDL-cholesterol, mmol/L | 1.59±0.43 | 1.55±0.41 | 1.51±0.40 | 1.45±0.38 | 1.53±0.41 |
| Family history of diabetes, % | 8.3 | 8.2 | 8.1 | 8.2 | 8.2 |
| Systolic blood pressure, mmHg | 126.4±15.6 | 126.3±15.4 | 126.0±15.2 | 125.7±15.0 | 126.1±15.3 |
| Diastolic blood pressure, mmHg | 74.7±9.3 | 74.9±9.4 | 74.9±9.4 | 75.0±9.5 | 74.9±9.4 |
| Hypertension, % |  |  |  |  |  |
| No | 69.6 | 71.9 | 73.0 | 73.7 | 72.1 |
| Borderline | 11.9 | 12.0 | 11.4 | 12.0 | 11.8 |
| Manifest | 18.5 | 16.0 | 15.6 | 14.3 | 16.1 |

*Data are expressed as unadjusted mean ± standard deviation for age, PROCAM diabetes risk value, fasting glucose, BMI, HDL-cholesterol, systolic blood pressure, and diastolic blood pressure; data are expressed as median (interquartile) for ultra-processed food intake (weight percentage); data are expressed as observed percentage for highest tertile of PROCAM diabetes risk algorithm, family history of diabetes, and hypertension.

Diabetes risk at baseline was calculated according to the PROCAM diabetes risk algorithm with the following formula [35]:

| *y* = | -18.5694 + 0.0258×age(years) + 6.461163×10^-3^×glucose (mmol/L) + 0.108×BMI (kg/m^2^)  - 0.4585×10^-3^×HDL-cholesterol (mmol/L) + 0.4190×family history of diabetes (no = 0;  yes = 1) + 0.1713×hypertension (no = 0; borderline = 1; manifest = 2) |
| --- | --- |
| Borderline hypertension was defined as: (1) systolic blood pressure ≥ 140 mmHg and < 160 mmHg; or (2) diastolic blood pressure ≥ 90 mmHg and < 95 mmHg. Manifested hypertension was defined as: (1) use of hypertensive medication; or (2) systolic blood pressure ≥ 160 mmHg; or (3) diastolic blood pressure ≥ 95 mmHg. | |

**Additional file 1: Table S3** - Relative contribution (weight percentage) of each food group to ultra-processed food consumption in diet

| **Ultra-processed food group** | **Mean** | **SD*** | **Median** | **25%**† | **75%**† |
| --- | --- | --- | --- | --- | --- |
| Staple/starchy food, and cereals | 23.0 | 9.0 | 22.1 | 16.7 | 28.3 |
| Dairy products (except cheese) | 15.2 | 10.7 | 13.7 | 7.2 | 20.9 |
| Sugary beverages | 13.3 | 13.1 | 9.7 | 2.9 | 20.3 |
| Other liquid foods | 7.7 | 11.6 | 2.4 | 0 | 11.10 |
| Sweets, candies, and other sugary products | 7.3 | 4.5 | 6.5 | 4.2 | 9.5 |
| Soup | 7.0 | 6.8 | 4.9 | 2.8 | 9.1 |
| Savory spreads, sauces, and salad toppings for bread | 4.8 | 3.1 | 4.2 | 2.6 | 6.3 |
| Cheese | 4.5 | 3.9 | 3.6 | 1.9 | 6.1 |
| Meat, fish | 4.3 | 3.1 | 3.8 | 2.3 | 5.7 |
| Ready-to-eat meals | 3.2 | 4.1 | 2.1 | 0 | 4.4 |
| Spreadable butter | 3.0 | 2.1 | 2.9 | 1.5 | 4.3 |
| Fast food | 2.6 | 2.8 | 1.9 | 0 | 3.9 |
| Sweet spreads | 2.3 | 2.3 | 1.7 | 0.5 | 3.3 |
| Savory snacks | 1.7 | 1.6 | 1.3 | 0.6 | 2.2 |
| Other solid foods | 0.1 | 0.5 | 0 | 0 | 0 |

*SD denotes standard deviation.

†Corresponding 25th or 75th percentile of consumption.

**Additional file 1: Table S4** - Complete case analysis of the associations between consumption of ultra-processed food, its consumption patterns, and incident type 2 diabetes*

(1) Total intake of ultra-processed food

|  | **Quartiles of ultra-processed food consumption** | | | |  |  |  |
| --- | --- | --- | --- | --- | --- | --- | --- |
|  | **First** | **Second** | **Third** | **Fourth** | **P-*trend*** | **Continuous†** | **P value** |
| Cases/population | 255/17 604 | 247/17 606 | 272/17 605 | 354/17 606 |  | 1128/70 421 |  |
| Incidence, % | 1.4 | 1.4 | 1.5 | 2.0 |  | 1.6 |  |
| Model 3‡ | 1 | 1.08 (0.90, 1.31) | 1.26 (1.03, 1.55) | 1.76 (1.41, 2.18) | <0.001 | 1.25 (1.16, 1.35) | <0.001 |
| Model 4‡ | 1 | 1.05 (0.86, 1.27) | 1.19 (0.97, 1.46) | 1.53 (1.22, 1.90) | <0.001 | 1.17 (1.09, 1.26) | <0.001 |

(2) Ultra-processed food consumption patterns

|  |  | **Quartiles of consumption pattern scores of ultra-processed food** | | | |  |  |  |
| --- | --- | --- | --- | --- | --- | --- | --- | --- |
| **Consumption patterns scores** | **Models‡** | **First** | **Second** | **Third** | **Fourth** | **P-*trend*** | **Continuous** | **P value** |
| Warm savory snack pattern | 3 | 1 | 1.11 (0.92, 1.33) | 1.24 (1.02, 1.51) | 1.44 (1.16, 1.78) | <0.001 | 1.15 (1.08, 1.23) | <0.001 |
|  | 4 | 1 | 1.06 (0.88, 1.27) | 1.12 (0.92, 1.37) | 1.18 (0.94, 1.47) | 0.131 | 1.07 (1.00, 1.15) | 0.058 |
| Traditional Dutch cuisine pattern | 3 | 1 | 1.04 (0.86, 1.25) | 1.14 (0.94, 1.39) | 1.09 (0.88, 1.36) | 0.307 | 1.05 (0.96, 1.14) | 0.268 |
|  | 4 | 1 | 1.02 (0.85, 1.23) | 1.11 (0.91, 1.34) | 1.05 (0.85, 1.31) | 0.514 | 1.03 (0.95, 1.11) | 0.524 |
| Sweet snack pattern | 3 | 1 | 0.71 (0.60, 0.84) | 0.60 (0.50, 0.72) | 0.60 (0.49, 0.74) | <0.001 | 0.83 (0.76, 0.90) | <0.001 |
|  | 4 | 1 | 0.77 (0.65, 0.92) | 0.68 (0.57, 0.82) | 0.71 (0.58, 0.88) | <0.001 | 0.87 (0.80, 0.95) | 0.002 |
| Cold savory snack pattern | 3 | 1 | 1.06 (0.88, 1.27) | 1.22 (1.02, 1.47) | 1.38 (1.15, 1.67) | <0.001 | 1.18 (1.11, 1.25) | <0.001 |
|  | 4 | 1 | 1.01 (0.84, 1.22) | 1.15 (0.96, 1.39) | 1.24 (1.03, 1.50) | 0.013 | 1.13 (1.06, 1.21) | <0.001 |

*In comparison to the main analysis, only results from model 3 and model 4 are shown, because model 1 and model 2 do not involve missing data.

†Continuous model indicates OR (95% CI) for an absolute increment of 10% consumption of ultra-processed food in the total diet.

‡Model 3: OR (95% CI) derived from multivariate logistic regression models adjusted for age, sex, Lifelines diet score, total energy intake, alcohol intake, smoking status, educational level, non-occupational moderate-to-vigorous physical activity level, and TV watching time, *n* = 64 917. Model 4: OR (95% CI) derived from multivariate logistic regression models adjusted for Model 3 covariates plus BMI, *n* = 64 902.

**Additional file 1: Table S5** - Associations between consumption of ultra-processed food, its consumption patterns, and incident type 2 diabetes, when excluding participants who were lost to follow-up after 24 months*

(1) Total intake of ultra-processed food

|  | **Quartiles of ultra-processed food consumption** | | | |  |  |  |
| --- | --- | --- | --- | --- | --- | --- | --- |
|  | **First** | **Second** | **Third** | **Fourth** | **P-*trend*** | **Continuous*** | **P value** |
| Cases/population | 224/15 035 | 220/15 261 | 240/15 169 | 308/15 126 |  | 992/60 591 |  |
| Incidence, % | 1.5 | 1.4 | 1.6 | 2.0 |  | 1.6 |  |
| Model 1† | 1 | 1.12 (0.93, 1.35) | 1.39 (1.16, 1.68) | 2.16 (1.80, 2.59) | <0.001 | 1.33 (1.25, 1.41) | <0.001 |
| Model 2‡ | 1 | 1.09 (0.90, 1.33) | 1.31 (1.06, 1.61) | 1.87 (1.51, 2.33) | <0.001 | 1.27 (1.18, 1.37) | <0.001 |
| Model 3§ | 1 | 1.09 (0.90, 1.33) | 1.30 (1.06, 1.59) | 1.81 (1.45, 2.25) | <0.001 | 1.25 (1.16, 1.35) | <0.001 |
| Model 4\|\| | 1 | 1.06 (0.87, 1.29) | 1.21 (0.98, 1.49) | 1.58 (1.27, 1.97) | <0.001 | 1.18 (1.09, 1.27) | <0.001 |

*Continuous model indicates OR (95% CI) for an absolute increment of 10% consumption of ultra-processed food in the total diet.

†Model 1: OR (95% CI) derived from multivariate logistic regression models adjusted for age and sex, *n* = 60 591.

‡Model 2: OR (95% CI) derived from multivariate logistic regression models adjusted for Model 1 covariates plus Lifelines diet score, total energy intake, and alcohol intake, *n* = 60 591.

§Model 3: OR (95% CI) derived from multivariate logistic regression models adjusted for Model 2 covariates plus smoking status, educational level, non-occupational moderate-to-vigorous physical activity level, and TV watching time, *n* = 60 588.

||Model 4: OR (95% CI) derived from multivariate logistic regression models adjusted for Model 3 covariates plus BMI, *n* = 60 575.

(2) Ultra-processed food consumption patterns

| **Consumption patterns scores** | **Models** | **Quartiles of consumption pattern scores of ultra-processed food** | | | |  |  |  |
| --- | --- | --- | --- | --- | --- | --- | --- | --- |
|  |  | **First** | **Second** | **Third** | **Fourth** | **P-*trend*** | **Continuous** | **P value** |
| Warm savory snack pattern | Cases/population | 258/15 274 | 243/15 128 | 232/15 149 | 259/15 040 |  | 992/60 591 |  |
|  | Model 1* | 1 | 1.19 (1.00, 1.43) | 1.39 (1.15, 1.69) | 1.89 (1.56, 2.30) | <0.001 | 1.23 (1.17, 1.29) | <0.001 |
|  | Model 2† | 1 | 1.17 (0.98, 1.41) | 1.35 (1.11, 1.65) | 1.84 (1.49, 2.27) | <0.001 | 1.24 (1.18, 1.31) | <0.001 |
|  | Model 3‡ | 1 | 1.12 (0.93, 1.34) | 1.23 (1.00, 1.50) | 1.52 (1.23, 1.89) | <0.001 | 1.17 (1.10, 1.24) | <0.001 |
|  | Model 4§ | 1 | 1.07 (0.89, 1.29) | 1.11 (0.90, 1.35) | 1.23 (0.99, 1.54) | 0.065 | 1.09 (1.01, 1.16) | 0.018 |
| Traditional Dutch cuisine pattern | Cases/population | 248/14 749 | 241/15 147 | 266/15 256 | 237/15 439 |  | 992/60 591 |  |
|  | Model 1* | 1 | 0.93 (0.78, 1.12) | 1.05 (0.89, 1.26) | 0.99 (0.83, 1.19) | 0.747 | 1.01 (0.95, 1.08) | 0.696 |
|  | Model 2† | 1 | 0.97 (0.80, 1.17) | 1.11 (0.91, 1.35) | 1.05 (0.84, 1.31) | 0.420 | 1.04 (0.96, 1.13) | 0.344 |
|  | Model 3‡ | 1 | 0.96 (0.79, 1.15) | 1.08 (0.89, 1.32) | 1.02 (0.82, 1.27) | 0.564 | 1.03 (0.95, 1.12) | 0.525 |
|  | Model 4§ | 1 | 0.95 (0.79, 1.15) | 1.03 (0.85, 1.25) | 0.98 (0.78, 1.21) | 0.960 | 1.00 (0.92, 1.09) | 0.938 |
| Sweet snack pattern | Cases/population | 344/14 974 | 246/15 072 | 204/15 196 | 198/15 349 |  | 992/60 591 |  |
|  | Model 1* | 1 | 0.71 (0.60, 0.83) | 0.59 (0.49, 0.70) | 0.60 (0.50, 0.71) | <0.001 | 0.82 (0.76, 0.89) | <0.001 |
|  | Model 2† | 1 | 0.69 (0.59, 0.82) | 0.56 (0.47, 0.67) | 0.53 (0.43, 0.65) | <0.001 | 0.78 (0.72, 0.86) | <0.001 |
|  | Model 3‡ | 1 | 0.72 (0.61, 0.85) | 0.60 (0.50, 0.73) | 0.59 (0.48, 0.72) | <0.001 | 0.82 (0.75, 0.89) | <0.001 |
|  | Model 4§ | 1 | 0.79 (0.67, 0.94) | 0.69 (0.58, 0.84) | 0.70 (0.57, 0.87) | <0.001 | 0.87 (0.80, 0.94) | 0.001 |
| Cold savory snack pattern | Cases/population | 246/14 960 | 235/15 158 | 261/15 255 | 250/15 218 |  | 992/60 591 |  |
|  | Model 1* | 1 | 1.02 (0.85, 1.22) | 1.15 (0.97, 1.39) | 1.14 (0.95, 1.36) | 0.074 | 1.09 (1.03, 1.16) | 0.005 |
|  | Model 2† | 1 | 1.09 (0.91, 1.31) | 1.28 (1.07, 1.54) | 1.30 (1.08, 1.58) | 0.002 | 1.14 (1.07, 1.21) | <0.001 |
|  | Model 3‡ | 1 | 1.12 (0.93, 1.34) | 1.35 (1.12, 1.62) | 1.41 (1.16, 1.70) | <0.001 | 1.16 (1.09, 1.23) | <0.001 |
|  | Model 4§ | 1 | 1.07 (0.89, 1.29) | 1.27 (1.05, 1.52) | 1.26 (1.03, 1.53) | 0.007 | 1.11 (1.04, 1.18) | 0.001 |

*Model 1: OR (95% CI) derived from multivariate logistic regression models adjusted for age and sex, *n* = 60 591.

†Model 2: OR (95% CI) derived from multivariate logistic regression models adjusted for Model 1 covariates plus Lifelines diet score, total energy intake, and alcohol intake, *n* = 60 591.

‡Model 3: OR (95% CI) derived from multivariate logistic regression models adjusted for Model 2 covariates plus smoking status, educational level, non-occupational moderate-to-vigorous physical activity level, and TV watching time, *n* = 60 588.

§Model 4: OR (95% CI) derived from multivariate logistic regression models adjusted for Model 3 covariates plus BMI, *n* = 60 575.

**Additional file 1: Table S6** - Associations between consumption of ultra-processed food and incident type 2 diabetes, using energy-adjusted ultra-processed food intake

(1) Residual method*

|  | **Quartiles of ultra-processed food consumption** | | | |  |
| --- | --- | --- | --- | --- | --- |
|  | **First** | **Second** | **Third** | **Fourth** | **P-*trend*** |
| Cases/population | 229/17 605 | 244/17 605 | 282/17 605 | 373/17 606 |  |
| Incidence, % | 1.3 | 1.4 | 1.6 | 2.1 |  |
| Model 1† | 1 | 1.15 (0.96, 1.38) | 1.46 (1.23, 1.74) | 2.23 (1.88, 2.64) | <0.001 |
| Model 2‡ | 1 | 1.08 (0.90, 1.30) | 1.30 (1.09, 1.56) | 1.84 (1.54, 2.21) | <0.001 |
| Model 3§ | 1 | 1.08 (0.90, 1.30) | 1.29 (1.08, 1.55) | 1.77 (1.48, 2.12) | <0.001 |
| Model 4\|\| | 1 | 1.03 (0.86, 1.24) | 1.19 (0.99, 1.43) | 1.48 (1.24, 1.78) | <0.001 |

(2) Per unit energy intake method (grams of ultra-processed food intake per 1000 kcal energy intake)¶

|  | **Quartiles of ultra-processed food consumption** | | | |  |
| --- | --- | --- | --- | --- | --- |
|  | **First** | **Second** | **Third** | **Fourth** | **P-*trend*** |
| Cases/population | 232/17 605 | 243/17 605 | 262/17 605 | 391/17 606 |  |
| Incidence, % | 1.3 | 1.4 | 1.5 | 2.2 |  |
| Model 1† | 1 | 1.13 (0.94, 1.36) | 1.34 (1.12, 1.61) | 2.31 (1.95, 2.73) | <0.001 |
| Model 2‡ | 1 | 1.08 (0.90, 1.30) | 1.22 (1.01, 1.47) | 1.93 (1.61, 2.31) | <0.001 |
| Model 3§ | 1 | 1.08 (0.90, 1.30) | 1.22 (1.01, 1.46) | 1.85 (1.54, 2.22) | <0.001 |
| Model 4\|\| | 1 | 1.05 (0.87, 1.27) | 1.12 (0.93, 1.36) | 1.57 (1.31, 1.89) | <0.001 |

*Residual method first calculated the unexplained residuals of ultra-processed food intake (grams/day) on total energy intake (kcal/day) using linear regression models. The derived residuals were then subsequently used as the predictor in logistic regression models to estimate the associations between ultra-processed food intake and incident type 2 diabetes.

†Model 1: OR (95% CI) derived from multivariate logistic regression models adjusted for age and sex, *n* = 70 421.

‡Model 2: OR (95% CI) derived from multivariate logistic regression models adjusted for Model 1 covariates plus Lifelines diet score, total energy intake, and alcohol intake, *n* = 70 421.

§Model 3: OR (95% CI) derived from multivariate logistic regression models adjusted for Model 2 covariates plus smoking status, educational level, non-occupational moderate-to-vigorous physical activity level, and TV watching time, *n* = 70 418.

||Model 4: OR (95% CI) derived from multivariate logistic regression models adjusted for Model 3 covariates plus BMI, *n* = 70 403.

¶Intake of ultra-processed food per unit energy intake (median[interquartile], grams/1000kcal): 270 (243-288), 330 (317-343), 389 (372-408), 493 (456-555), and 358 (303-429), for quartile 1, quartile 2, quartile 3, quartile 4, and total population, respectively.

**Additional file 1: Table S7** - Consumption patterns of ultra-processed food and corresponding factor loadings derived from principle component analysis

| **Food items** | **Warm savory**  **snack pattern** | **Traditional**  **Dutch pattern** | **Sweet**  **snack pattern** | **Cold savory**  **snack pattern** |
| --- | --- | --- | --- | --- |
| Sliced bread |  | 0.4770 |  |  |
| Cream cheese, foreign cheese |  |  |  | 0.3113 |
| Ham sausages, bacon slices (bread) |  | 0.3041 |  |  |
| Other types of processed meat (for bread) |  | 0.2833 | -0.2014 |  |
| Chocolate spreads |  |  | 0.2767 |  |
| Other types of sweet spreads |  |  | 0.2402 |  |
| Full-fat custard |  | 0.2072 |  |  |
| Meals from fast-food restaurants | 0.2202 |  |  |  |
| Fries | 0.2762 |  |  |  |
| Gravy |  | 0.2897 |  |  |
| Warm sauce (pasta sauce, mushroom sauce, sate sauce) |  |  |  | 0.2103 |
| Mayonnaise | 0.2081 |  |  |  |
| Low fat mayonnaise, sauce for fries,  non-red sauce | 0.2321 |  |  |  |
| Cheese with warm meals |  |  |  | 0.3368 |
| Small cookies/biscuits |  |  | 0.3981 |  |
| Pastries |  |  | 0.2965 |  |
| Cakes |  |  | 0.2403 |  |
| Candy bars |  |  | 0.2086 |  |
| Chocolates |  |  | 0.3244 |  |
| Candies |  |  | 0.2271 |  |
| Warm savory snacks (croquettes, minced meat hotdogs, sausage rolls) | 0.4358 |  |  |  |
| Mayonnaise (for snacks) | 0.3053 |  |  |  |
| Low fat mayonnaise, sauce for fries, non-red sauce (for snacks) | 0.3330 |  |  |  |
| Snack cheese |  |  |  | 0.3596 |
| Ham sausages, sliced deli meat as snacks |  |  |  | 0.2683 |
| Salad (spreads) on bread/baguettes, sandwich spreads |  |  |  | 0.2515 |
| Sugary beverages, lemonade with sugar | 0.2184 |  |  |  |
| Spreadable butter, margarine |  | 0.2145 |  |  |
| Low-fat margarine |  | 0.2062 |  |  |
| **Eigenvalues*** | 4.05 | 2.38 | 1.98 | 1.66 |
| **Explained Variance %** | 4.96 | 3.84 | 3.43 | 3.27 |
| **Cumulative Explained Variance %** | 4.96 | 8.80 | 12.23 | 15.50 |

*From the fifth component (eigenvalue = 1.56) and the following, the differences in eigenvalues substantially dropped (≤0.1).

**Additional file 1: Table S8** - Baseline characteristics of study participants according to the highest quartiles of ultra-processed food consumption pattern scores (*n* = 70 421)*

|  | **Highest quartiles of ultra-processed food consumption pattern scores** | | | |  |
| --- | --- | --- | --- | --- | --- |
|  | **Warm savory snack pattern** | **Traditional Dutch cuisine pattern** | **Sweet snack pattern** | **Cold savory snack pattern** | **Total (*n* = 70 421)** |
| Sample size | 292 | 269 | 224 | 281 | 1128 |
| Type 2 diabetes cases | 17 606 | 17 606 | 17 606 | 17 606 | 70 421 |
| Type 2 diabetes incidence, % | 1.66 | 1.53 | 1.27 | 1.60 | 1.60 |
| Age, years | 45.2±6.8 | 48.2±8.5 | 48.1±8.7 | 48.5±8.3 | 49.1±8.8 |
| Sex, %: |  |  |  |  |  |
| Women | 59.4 | 59.4 | 58.9 | 58.5 | 58.6 |
| Men | 40.6 | 40.6 | 41.1 | 41.5 | 41.4 |
| Ultra-processed food intake, weight% | 41.5 (34.5, 49.1) | 37.5 (31.4, 44.5) | 38.0 (31.3, 45.3) | 36.9 (30.2, 44.5) | 34.9 (28.1, 42.7) |
| Lifeline diet score | 21.0±5.2 | 22.3±5.5 | 22.6±5.6 | 22.9±5.7 | 24.0±5.9 |
| Total energy intake, kcal/day | 2350±638 | 2483±617 | 2432±594 | 2306±622 | 2063±598 |
| Total alcohol intake, grams/day | 4.0 (0.8, 11.6) | 3.6 (0.8, 10.0) | 2.9 (0.6, 8.7) | 6.9 (2.6, 15.2) | 4.7 (0.9, 11.2) |
| Fasting glucose, mmol/L | 4.97±0.50 | 4.94±0.50 | 4.91±0.49 | 4.99±0.51 | 4.96±0.50 |
| HbA_1c_, % | 5.53±0.30 | 5.56±0.29 | 5.55±0.29 | 5.54±0.30 | 5.55±0.30 |
| BMI, kg/m^2^ | 26.8±4.4 | 26.1±4.2 | 25.7±4.0 | 26.2±4.1 | 26.2±4.1 |
| Highest tertile of PROCAM diabetes risk algorithm, % | 29.7 | 30.6 | 27.3 | 31.6 | 33.2 |
| MVPA, minutes/week† | 150 (50, 300) | 180 (60, 360) | 190 (75, 364) | 200 (75, 375) | 190 (60, 365) |
| Educational level, %: |  |  |  |  |  |
| Low | 33.8 | 33.9 | 26.3 | 25.0 | 30.4 |
| Middle | 44.0 | 41.3 | 40.6 | 37.7 | 38.9 |
| High | 22.0 | 24.3 | 32.7 | 37.1 | 30.3 |
| Smoking status, %: |  |  |  |  |  |
| Never | 42.7 | 46.7 | 52.7 | 40.9 | 44.4 |
| Former | 32.8 | 35.2 | 33.4 | 39.6 | 37.9 |
| Current | 23.9 | 17.5 | 13.3 | 18.8 | 17.2 |
| TV watching time, hours/day | 2.7±1.3 | 2.4±1.3 | 2.4±1.3 | 2.4±1.3 | 2.5±1.3 |

*Data are expressed as unadjusted mean ± standard deviation for age, Lifelines diet score (no unit), total energy intake, fasting glucose, HbA1c, BMI, and TV watching time; data are expressed as median (interquartile) for ultra-processed food intake (weight percentage), total alcohol intake, and MVPA; data are expressed as observed percentage for sex, highest tertile of PROCAM diabetes risk algorithm, educational level, and smoking status.

†MVPA denotes non-occupational moderate-to-vigorous physical activity level.

**Additional file 1: Table S9** - Consumption patterns of ultra-processed food and corresponding factor loadings derived from principle component analysis, sensitivity analysis using random half samples*

| **Food items** | **Warm savory**  **snack pattern** | **Traditional**  **Dutch pattern** | **Sweet**  **snack pattern** | **Cold savory**  **snack pattern** |
| --- | --- | --- | --- | --- |
| Sliced bread |  | 0.4726^1^; 0.4711^2^; 0.4738^3^ |  |  |
| Cream cheese, foreign cheese |  |  |  | 0.3169^1^; 0.3207^2^; 0.3048^3^ |
| Ham sausages, bacon slices (bread) |  | 0.3007^1^; 0.3059^2^; 0.2914^3^ |  |  |
| Other types of processed meat (for bread) |  | 0.2853^1^; 0.2901^2^; 0.2704^3^ | -0.1950^1^; -0.1853^2^; -0.1970^3^ |  |
| Chocolate spreads |  | 0.1909^1^; 0.1771^2^; 0.2028^3^ | 0.2729^1^; 0.2797^2^; 0.2679^3^ |  |
| Other types of sweet spreads |  |  | 0.2419^1^; 0.2402^2^; 0.2336^3^ |  |
| Full-fat custard |  | 0.2032^1^; 0.2024^2^; 0.2116^3^ |  |  |
| Meals from fast-food restaurants | 0.2241^1^; 0.2179^2^; 0.2238^3^ |  |  |  |
| Fries | 0.2808^1^; 0.2768^2^; 0.2789^3^ |  |  |  |
| Gravy |  | 0.2911^1^; 0.2967^2^; 0.2927^3^ |  |  |
| Warm sauce (pasta sauce, mushroom sauce, sate sauce) |  |  |  | 0.2004^1^; 0.2022^2^; 0.1952^3^ |
| Mayonnaise | 0.2057^1^; 0.2067^2^; 0.2039^3^ |  |  |  |
| Low fat mayonnaise, sauce for fries,  non-red sauce | 0.2251^1^; 0.2396^2^; 0.2463^3^ |  |  |  |
| Cheese with warm meals |  |  |  | 0.3305^1^; 0.3316^2^; 0.3267^3^ |
| Small cookies/biscuits |  |  | 0.3991^1^; 0.3985^2^; 0.4016^3^ |  |
| Pastries |  |  | 0.2894^1^; 0.2899^2^; 0.2903^3^ |  |
| Cakes |  |  | 0.2509^1^; 0.2425^2^; 0.2348^3^ |  |
| Breakfast/nutrition biscuits |  |  | 0.2033^1^; 0.1954^2^; 0.2008^3^ |  |
| Candy bars |  |  | 0.2131^1^; 0.1992^2^; 0.2221^3^ |  |
| Chocolates |  |  | 0.3207^1^; 0.3219^2^; 0.3312^3^ |  |
| Candies |  |  | 0.2230^1^; 0.2363^2^; 0.2213^3^ |  |
| Warm savory snacks (croquettes, minced meat hotdogs, sausage rolls) | 0.4305^1^; 0.4344^2^; 0.4295^3^ |  |  |  |
| Mayonnaise (for snacks) | 0.3013^1^; 0.3027^2^; 0.2928^3^ |  |  |  |
| Low fat mayonnaise, sauce for fries, non-red sauce (for snacks) | 0.3221^1^; 0.3360^2^; 0.3326^3^ |  |  |  |
| Chips | 0.2103^1^; 0.2013^2^; 0.1833^3^ |  |  |  |
| Snack cheese |  |  |  | 0.3460^1^; 0.3553^2^; 0.3806^3^ |
| Ham sausages, sliced deli meat as snacks |  |  |  | 0.2594^1^; 0.2632^2^; 0.2858^3^ |
| Salad (spreads) on bread/baguettes, sandwich spreads |  |  |  | 0.2331^1^; 0.2477^2^; 0.2700^3^ |
| Sugary beverages, lemonade with sugar | 0.2192^1^; 0.2264^2^; 0.2227^3^ |  |  |  |
| Spreadable butter, margarine |  | 0.2120^1^; 0.1956^2^; 0.2082^3^ |  | 0.2085^1^; 0.1863^2^; 0.1974 |
| Low-fat margarine |  | 0.2090^1^; 0.2165^2^; 0.2058^3^ |  |  |
| **Eigenvalues** | 4.03^1^; 4.05^2^; 4.01^3^ | 2.38^1^; 2.39^2^; 2.39^3^ | 1.98^1^; 1.99^2^; 2.01^3^ | 1.67^1^; 1.65^2^; 1.65^3^ |
| **Explained Variance %** | 4.97^1^; 4.90^2^; 4.94^3^ | 3.84^1^; 3.86^2^; 3.89^3^ | 3.41^1^; 3.46^2^; 3.42^3^ | 3.24^1^; 3.29^2^; 3.21^3^ |
| **Cumulative Explained Variance %** | 4.97^1^; 4.90^2^; 4.94^3^ | 8.82^1^; 8.76^2^; 8.82^3^ | 12.23^1^; 12.23^2^; 12.25^3^ | 15.47^1^; 15.52^2^; 15.46^3^ |

*Superscripts denotes each random half sample.

**Additional file 1: Table S10** - Complete case analysis of the associations of ultra-processed food intake and its consumption patterns with type 2 diabetes risk at baseline*

| **Ultra-processed food consumption (patterns)** | **Standardized beta-coefficients**† |
| --- | --- |
| Total ultra-processed food intake | 0.051 (0.044, 0.059) |
| Warm savory snack pattern | 0.091 (0.082, 0.100) |
| Traditional Dutch cuisine pattern | -0.033 (-0.042, -0.024) |
| Sweet snack pattern | -0.102 (-0.111, -0.092) |
| Cold savory snack pattern | 0.042 (0.032, 0.051) |

*Type 2 diabetes risk at baseline was assessed by PROCAM diabetes risk algorithm (Supplementary Table S2).

†Standardized beta-coefficients (95% CI) derived from multivariate linear regression models adjusted for age, sex, Lifelines diet score, alcohol intake, smoking status, educational level, non-occupational moderate-to-vigorous physical activity level, and TV watching time, all P values < 0.001, *n* = 64 650 .
